# Self-Construal Priming Affects Speed of Retrieval from Short-Term Memory

**DOI:** 10.1371/journal.pone.0050007

**Published:** 2012-11-29

**Authors:** Justin A. MacDonald, Joshua Sandry, Stephen Rice

**Affiliations:** Department of Psychology, New Mexico State University, Las Cruces, New Mexico, United States of America; CSIC-Univ Miguel Hernandez, Spain

## Abstract

We investigated the effects of collective or individual self-construal priming on recall in a short-term memory (STM) task. We primed participants to either their individual or their collective self-construals or a neutral control condition. Participants then completed a STM retrieval task using either random or patterned digit strings. Findings revealed that priming an individual self-construal resulted in faster retrieval of information from STM for both stimulus types. These results indicate that individual self-accessibility improves retrieval speed of digits from STM, regardless of set configuration. More broadly, the present findings extend prior research by adding further evidence of the effects of self-construal priming on cognitive information processing.

## Introduction

A person's construal of the ‘self’ can have a fundamental impact on the entire experience of that individual [1]. One common classification of self-construals that has been used to explain differences in social norms and behaviors across cultures is the distinction between independent and interdependent self-construals [1], [2], [3]. Those with independent (or individualistic) self-construals tend to define themselves based on their individual or personal traits, and people with interdependent (or collectivistic) self-construals tend to define themselves within the context of a larger community [1], [4]. Of course, members of a collectivistic culture will vary in their level of collectivism just as those in an individualistic culture will vary in their level of individualism. Rather than operating with a single self-construal that lies at some point along a collectivistic/individualistic continuum, however, individuals possess both individualistic and collectivistic self-construals simultaneously, and the dominance of one over the other can be manipulated through priming methods [4], [5]. The effectiveness of these priming techniques in eliciting effects has been demonstrated repeatedly in a variety of research domains, including attitudes and beliefs [6], [7], consumer behavior [8], and social comparisons [9], [10].

Given the far-reaching impact that self-construals are thought to have, manipulating them should result in effects well beyond those behaviors directly related to individualism or collectivism. Recent research has demonstrated self-construal priming effects in visual search behavior [11], [12] and visual pattern recognition [13]. Oyserman et al. [13], [ see also 14] demonstrated that priming individual or collective self-construals results in differential processing of both visual and auditory stimuli. Priming individual self-construals enhanced processing of separate stimulus components, and priming a collective self-construal enhanced processing of the overall stimulus structure [13], [15]. In a series of eight studies, Oyserman and colleagues demonstrated that collective priming results in better performance in tasks that require attending to the overall stimulus structure and worse performance in tasks that are facilitated by ignoring parts of the stimulus [13]. For example, collectively-primed participants performed more slowly under accuracy instructions and less accurately under time pressure when completing a task that required ignoring part of an auditory stimulus. In addition, Lin and Han [16] presented subjects with matrices of alphabetical letters that included a large (‘global’) letter constructed from instances of a small (‘local’) letter (e.g., a large ‘S’ constructed from small ‘A’'s). When this stimulus was presented in a matrix with distractors, individually-primed participants were faster than collectively- and neutrally-primed participants at identifying the local letter, and collectively-primed participants were the fastest at identifying the global letter [16], [ see also 14].

Several researchers have demonstrated self-construal priming effects in long-term memory (LTM) as well. Wang and Ross [17] reported self-priming effects on retrieval of autobiographical memories from LTM. Participants that received the collective prime tended to retrieve memories of routine social situations of a collective nature, and those that received the individual prime tended to retrieve events centered on themselves. Sui, Zhu, and Chiu [18] examined the effects of cultural priming on recognition memory using a sample of Chinese participants. Subjects were primed with images representative of Chinese or American culture, which effectively served as collective and individual primes, respectively. The participants were then shown a series of adjectives and asked to rate each in terms of its applicability to either the subject or the subject's mother. After a one-hour delay, participants completed a surprise recognition task in which they viewed a list of adjectives and indicated which were encountered during the rating task. Participants in the Chinese prime condition showed significantly better recognition of the mother-relevant adjectives, and participants in the American prime condition demonstrated better recognition of the self-relevant adjectives.

The results of Wang and Ross [17] and Sui et al. [18] indicate that the effects of self-construal priming extend into memory processes. In a sense, these results are surprising given the conceptual distance from social self-construals to LTM processes. Considering the wide-reaching impact of self-construals, however, the results are perhaps less surprising. Both the Wang and Ross [17] and Sui et al. [18] memory studies used methodologies that allow for (and arguably encourage) the encoding and retrieval of each to-be-remembered item within a context that is closely related to collectivism or individualism. For example, the autobiographical memories considered in the Wang and Ross [17] study are episodic in nature, and therefore they consist of memories of the individual, either alone or in a social context. The participants in the Sui et al. [18] study were told to apply the to-be-remembered adjectives to either themselves (individual) or their mother (collective). Therefore, it is not particularly surprising that self-construal priming affected these processes, as the link between self-construal and LTM encoding and retrieval in these experiments was direct.

The purpose of this brief report is to describe the results of a riskier endeavor: an effort to find effects of self-construal priming on retrieval from short-term memory (STM), using to-be-remembered stimuli that are less directly linked to individualism and collectivism. The point of the study was to identify self-construal priming effects on the memory processes themselves rather than effects due to the memory content. We used the self-construal primes (neutral, individual, and collective versions) from Trafimow et al. [4] due to their widespread use in a variety of experimental paradigms [8], [19], [20]. These primes take the form of a short story about an ancient Sumerian warrior that makes a decision about whom to put in charge of part of his army. In the collective version of the prime, the warrior makes this decision based on family considerations, and in the individual prime the warrior makes this decision based on the benefits to the warrior himself. In the neutral version of the prime no reason is given for the decision. The complete primes are included in Appendix S1. We chose a digit recall task to measure STM performance because digits are less likely to be encoded or retrieved with information that is individualistic or collectivistic in nature. On each trial, participants were shown a number stimulus that consisted of 3–12 digits. After stimulus offset, participants were asked to respond with the *n*th digit of the stimulus, where *n* ranged from 1 to the number of digits in the stimulus. We included two different configurations of digits in the experiment as well: half of the number stimuli had an obvious pattern (e.g., 111222333) and half were random sequences of digits. Due to the exploratory nature of this study, priming effects of any kind were of interest. We put forward two specific hypotheses, however: collective priming should lead to decreased RTs relative to individual priming for patterned stimuli, and individual priming should lead to lower RTs relative to collective priming for random stimuli.

## Methods

### Participants

Ninety-five participants (33 males, 62 females, M_age_ = 20.50 years, SD = 3.90) from introductory psychology classes at New Mexico State University volunteered to participate in this study for course credit. This research was approved by the New Mexico State University Institutional Review Board. Participants provided written informed consent.

### Materials and Procedure

After reading and signing an informed consent form, participants read a story about a warrior who had to choose the commander of an army. The individual and collective priming conditions added a description of how his decision benefitted either his own self-interest or his family's interest, respectively (see [4]). Next, participants listed five benefits of the warrior's decision (in the individual and collective conditions) or five events from their day (in the neutral control condition), taken from Rice et al. [11]. The purpose of this was to increase the strength of the priming effect and to make sure that the participant read the prime (see Appendix S1). Finally, participants performed 150 trials in which they viewed a string of either patterned or randomly-organized digits (set sizes ranged between three to 12 digits) and were then asked to retrieve a digit from a randomly selected location within the set. Digits were randomly ordered (e.g. 591263) in the Random condition and had an obvious pattern (e.g., 897897) in the Patterned condition. All trials consisted of a digit string presented for 3000 ms, followed by a fixation cross presented for 500 ms, followed by a probe screen asking participants to enter a particular digit from the string (e.g. the fifth digit). Feedback was provided. Prime type was varied randomly across subjects, and digit configuration was randomized across trials.

## Results and Discussion

**Figure 1:**
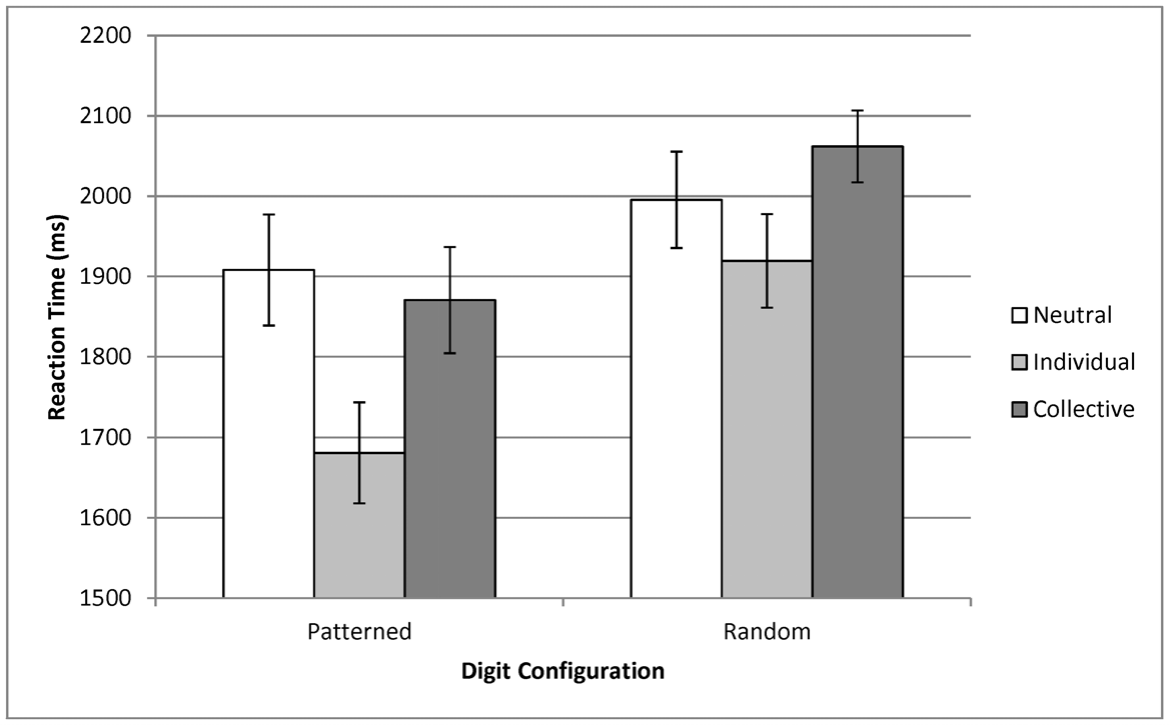
Mean RT as a function of Digit Configuration and Prime Type. Each bar represents the mean RT for one of the six Digit Configuration/Prime combinations. The bars around each mean indicate the standard error.

Accuracy rates were nearly identical across priming conditions. Mean proportions correct in the Patterned condition were 0.92, 0.91, and 0.92 in the Neutral/Patterned, Individual/Patterned, and Collective/Patterned cells, respectively; mean proportions correct in the Neutral/Random, Individual/Random, and Collective/Random cells were 0.76, 0.75, and 0.76, respectively. This constant accuracy across priming conditions is consistent with the results of the studies in Oyserman et al. [13] that did not introduce time pressure. Mean RTs were calculated for each participant and digit configuration condition after discarding incorrect trials. RTs shorter than 300 ms or greater than three standard deviations above the mean for that participant and condition were excluded from the analyses; this resulted in the exclusion of 2.9% of correct trials. Mean RTs for all conditions are shown in Figure 1. The results of our two-tailed planned comparisons indicated that the Collective/Patterned (*M* = 1870 ms, *SD* = 374 ms) and Individual/Patterned (*M* = 1681 ms, *SD* = 350 ms) conditions were significantly different, *t*(61) = 2.08, p = 0.042, *d* = 0.52, although this difference was in the opposite direction than expected. The difference between the Collective/Random (*M* = 2062 ms, *SD* = 253 ms) and Individual/Random (*M* = 1919 ms, *SD* = 324 ms) conditions was marginally significant and in the expected direction, *t*(61) = 1.95, p = 0.056, *d* = 0.48, with a meaningful effect size. Testing for effects of the individual and collective primes relative to the neutral, Individual/Patterned RTs were significantly different from Neutral/Patterned RTs, *t*(61) = 2.42, p = 0.018, *d* = 0.60. All other comparisons to the control were insignificant.

Our predictions were only partially confirmed in this study. Unexpectedly, individual priming led to *decreased* RTs relative to collective priming in both the Patterned and Random conditions. This result was expected in the Random condition, as we expected the improvement in local processing resulting from the individual prime to improve performance with a randomly-ordered stimulus, and the enhanced global processing arising from collective priming should not be useful in this situation due to the lack of any pattern in the stimulus. However, in the Patterned condition we hypothesized that enhanced global processing would lead to faster recognition of the patterns in the stimulus, thereby decreasing the overall RT, but this effect did not occur. In hindsight, it appears likely that this result is a consequence of the task used in our experiment. This task requires the participant to ignore any pattern in the stimulus, instead focusing on the retrieval of a single digit. The results of Oyserman et al. [13] indicate that collectively-primed participants are less able to ignore part of a stimulus. In Study 6 of that paper, participants were presented with a speech stream to each of the ears and were asked to attend selectively to just one of them. Individually-primed participants exhibited faster performance than those exposed to the collective prime, presumably because Individual priming enhanced the ability to disregard part of the auditory stimulus. In the Patterned condition in our study, individually-primed participants were able to ignore the irrelevant overall pattern in the stimulus, thereby performing faster at digit recall than those in the collective and neutral prime conditions. This result coincides neatly with those of Oyserman et al. [13]. This explanation would also account for the improved performance of the individually-primed participants in the Random condition: the task requires ignoring the majority of the stimulus to focus on a single digit, and collective priming should impair this ability.

In summary, the results of this small study clearly indicate that self-construal priming affects performance on a STM retrieval task. Our results correspond well with those of Oyserman et al. [13] despite the differences in prime, stimulus modality, and dependent variable. Our findings extend prior literature by further demonstrating how self-construal priming directly impacts cognitive processing. Our results are all the more noteworthy given the conceptual distance between self-construals about social behavior and the cognitive processing of digit stimuli in STM. Whether priming effects manifest in the encoding, storage, rehearsal, or retrieval phases of the STM task remains an open question, one that should be tested in a future study. Given that the effects on RT were measured during the retrieval phase, retrieval seems the most likely component of STM to be affected by self-construal priming in the present study. The next step in this research program is to identify STM tasks for which collective self-construal priming leads to enhanced performance relative to individual and neutral priming.

## Supporting Information

Appendix S1 **Priming manipulations used in the experiment.** These primes are originally from [4].
(DOCX)
