# Supplementary material for: Self-Construal Priming Affects Speed of Retrieval from Short-Term Memory
Source: PLoS One. 2012 Nov 29;7(11):e50007. doi: 10.1371/journal.pone.0050007 (PMC3510200; doi:10.1371/journal.pone.0050007)
Supplement: Appendix S1 — Priming manipulations used in the experiment. These primes are originally from [4]. (DOCX) [file pone.0050007.s001.docx]

Appendix S1

Experiment # _______

Participant # _______

**Please read the following short story and complete the tasks on the following page.**

Sostoras, a warrior in ancient Sumer, was largely responsible for the success of Sargon I in conquering all of Mesopotamia. As a result, he was rewarded with a small kingdom of his own to rule.

About 10 years later, Sargon I was conscripting warriors for a new war. Sostoras was obligated to send a detachment of soldiers to aid Sargon I. He had to decide whom to put in command of the detachment. After thinking about it for a long time, Sostoras eventually decided on Tiglath, who was a member of his family. This appointment had several advantages. Sostoras was able to show his loyalty to his family. He was able to cement their loyalty to him. In addition, having Tiglath as the commander increased the power and prestige of the family. Finally, if Tiglath performed well, Sarson I would be indebted to the family.

Please turn the page and complete the tasks.

List five ways in which Sostoras’ actions could potentially help his family.

1. ______________________________________________________________________________
2. ______________________________________________________________________________
3. ______________________________________________________________________________
4. ______________________________________________________________________________
5. ______________________________________________________________________________

Experiment # _______

Participant # _______

**Please read the following short story and complete the tasks on the following page.**

Sostoras, a warrior in ancient Sumer, was largely responsible for the success of Sargon I in conquering all of Mesopotamia. As a result, he was rewarded with a small kingdom of his own to rule.

About 10 years later, Sargon I was conscripting warriors for a new war. Sostoras was obligated to send a detachment of soldiers to aid Sargon I. He had to decide whom to put in command of the detachment. After thinking about it for a long time, Sostoras eventually decided on Tiglath, who was a talented general indebted to him. This would solidify Sostaras’ hold on his own dominion. In addition, the very fact of having a general such as Tiglath as his personal representative would increase Sostoras’ prestige. Finally, sending his best general would be likely to make Sargon I grateful. Consequently, there was the possibility of getting rewarded by Sargon I.

Please turn the page and complete the tasks.

List five ways in which Sostoras’ actions could potentially help him.

1. ______________________________________________________________________________
2. ______________________________________________________________________________
3. ______________________________________________________________________________
4. ______________________________________________________________________________
5. ______________________________________________________________________________

Experiment # _______

Participant # _______

**Please read the following short story and complete the tasks on the following page.**

Sostoras, a warrior in ancient Sumer, was largely responsible for the success of Sargon I in conquering all of Mesopotamia. As a result, he was rewarded with a small kingdom of his own to rule.

About 10 years later, Sargon I was conscripting warriors for a new war. Sostoras was obligated to send a detachment of soldiers to aid Sargon I. He had to decide whom to put in command of the detachment. After thinking about it for a long time, Sostoras eventually decided on Tiglath.

Please turn the page and complete the tasks.

List five things you plan to do today.

1. ______________________________________________________________________________
2. ______________________________________________________________________________
3. ______________________________________________________________________________
4. ______________________________________________________________________________
5. ______________________________________________________________________________
